# Motorized and non-motorized mixed traffic characteristics and lateral opening spacing calculation for the section of arterial highway through small towns

**DOI:** 10.1038/s41598-024-55529-0

**Published:** 2024-02-28

**Authors:** Shengneng Hu, Kexin Mao, Wei Tong, Zhen Jia, Weibo Zhai, Hongwei Xu

**Affiliations:** 1 Ural Institution, North China University of Water Resources and Electric Power, Zhengzhou, 450011 China; 2 School of Civil Engineering and Communication, North China University of Water Resources and Electric Power, Zhengzhou, 450011 China; 3 Electric Engineering Company of China Railway Seventh Group, Zhengzhou, 450011 China

**Keywords:** Transportation environment, Highway through small town section, Spacing between crossing openings, Road planning, Engineering, Civil engineering

## Abstract

A large number of motor vehicles and non-motorized vehicles mixed in the section of arterial highway crossing the town leads to many traffic problems. Therefore, it is necessary to set up a side divider between motorized and non-motorized lanes, and the appropriate spacing of lateral crossing openings to meet the needs of non-motorized vehicles crossing the highway has become a key issue that must be resolved. This paper investigates the traffic flow characteristics of mixed traffic flow on arterial highways through small town sections, and from the two dimensions of highway access efficiency and the psychological characteristics of cyclists, it calibrates the setting range of the spacing of non-motorized lateral crossing openings under different design speeds, which is used to regulate the behavior of non-motorized vehicles crossing the street, improve the safety level of the highway, reduce the lateral interference of the highway, and improve the road access efficiency. The accuracy of the research results is verified by microscopic simulation experiments, which proves that they can meet good expectations in practical engineering. The research results have theoretical significance and reference value for improving the status quo of machine-non-mixed traffic in the section of arterial highway passing through small towns, and enhancing the efficiency and safety of highway traffic. It also provides corresponding reference for the areas facing similar problems worldwide.

## Introduction

The section of arterial highway through small towns is a special environmental section due to the gathering of population and various production factors to both sides of the highway, resulting in the development of the highway along the road in a ribbon development. This type of roadway not only assumes the function of passing traffic, but also serves as a link between communities along the street, and has the dual characteristics of an urban road and a highway^1^. This type of road section is designed to serve mainly motor vehicles, but due to the dense residential areas on both sides of the road, pedestrians, non-motorized vehicles and motor vehicles mixed, complex traffic, easy to produce the road's functional settings to meet the contradictions of traffic demand, resulting in traffic congestion, traffic accidents, road capacity significantly reduced and other issues^2^. As this type of road section has been built and put into use in accordance with the design standards of the highway single width road, therefore, the optimal way to solve the traffic problems of such road sections, to avoid the mixing of different nature traffic flow, the road cross-section to increase the side split zone, the physical separation of motorized and non-motorized traffic flow. Therefore, to meet the needs of non-motorized vehicles crossing the road and set the spacing of the lateral crossing openings, it becomes a key issue that must be resolved.

Due to differences in national conditions, the size of small towns and the number of non-motorized vehicles in each country are far less than in China^3^. At the same time, many countries have restrictions at the legal level on the passage of non-motorized vehicles on roads^4^. For example, in the United States, New York and Canada, traffic regulations prohibit non-motorized vehicles from driving on fast lanes, main roads, and interstate highways unless there is a permit sign^5,6^. Therefore, the traffic problems caused by mixing motorized and non-motorized vehicles on arterial roads are not as obvious as in China. And foreign scholars have focused their research on road intersection settings, rural road safety, and non-motorized vehicle safety. The U.S. has conducted a systematic study of road intersections, developed an intersection management manual, and conducted key technical studies on road intersection spacing, road median openings, U-turn settings, and roadways along streets, respectively. Praticò F G and Giunta M analyzed the influence of road characteristics and traffic environment on driver behavior and travel speed on a two-lane road through a township, concluding that factors such as road curve radius, intersection density and speed limit management significantly affect motor vehicle speed^7^. Petzoldt T also pointed out that the difference in the probability of being involved in a traffic conflict between a human-powered bicycle and an electric bicycle on a normal roadway is not significant, but at an intersection, an electric bicycle is twice as likely to be involved in a conflict as a human-powered bicycle^8^. Saeed Garmei and Ehsan Kashi simulated various parameters of pedestrians and vehicles using Aimsun to analyze the impact of pedestrians on the capacity of roundabout intersections^9^. Chinese scholars have more abundant research on arterial highways through small town sections, electric vehicle riding patterns, mixed traffic flow characteristics, and urban road intersections. He Yulong et al. put forward the concept of design and traffic stabilization of the gradual change section of highway through villages and towns, which provided ideas for later scholars to solve the safety problems of the section of arterial highway through villages and towns^10^. Chen Xu researched some key technical indicators such as the longitudinal section and the number of lanes in the transition section of the trunk highway in China's village-town area and proposed the formula and recommended values for the length and gradual change rate of the transition section of the road articulation in the village-town area based on the lane change trajectory model^11^. Hu Shengneng studied the spatial and temporal distribution and traffic flow parameters of non-motorized vehicles in road-crossing townships to obtain the rules and characteristics of non-motorized traffic in terms of travel purpose, travel distance, travel time and travel frequency and mastered the travel pattern of non-motorized cyclists in road crossing townships^12^. Lei Xueqi used regression curves to analyze the motion characteristics of the mixed flow of conventional bicycles and electric bicycles in urban non-motorized lanes, pointing out that the proportion of electric bicycles and the width of non-motorized lanes have a large impact on the operating speed and density of the mixed non-motorized flow, and giving the recommended values of non-motorized lane width for different road conditions and the proportion of mixed electric bicycles^13^. Cao Rongqing studied the relationship between the intersection spacing of urban roads and the number of traffic conflict points, accident rate and capacity, and established a minimum spacing model between two adjacent intersections^14^.

Scholars in China and abroad have conducted a lot of research at road intersections, and many research results have been achieved, but the research on the spacing of non-motorized crossing openings for arterial highways through small town sections is almost blank. Non-motorized traffic flow within the road through small towns has specific rules and characteristics, and non-motorized traffic accidents at intersections are high and frequent. Relevant research results from the perspective of motor vehicle demand, based on the road network layout structure, to determine the spacing of urban road intersections, the conclusions of the study do not apply to arterial highways through town sections. Therefore, this paper analyzes the mixed traffic flow characteristics of arterial highway sections through small towns, and constructs a model for calculating the spacing of non-motorized crossing openings in the side-separation zone based on the separation of machine and non-machine, so as to quantitatively calculate the reasonable spacing of non-motorized crossing openings.

## Traffic survey methods

### Survey site selection

The sampling principles for the survey sites were as follows:

1. Road grade is secondary road;
2. The road crosses the village and town area;
3. Motor vehicles and non-motorized vehicles are mixed, and there is no physical separation of road cross-sections;
4. The topography of the road section is gentle, and the longitudinal slope of the road is a gentle slope section.

**Figure 1:**
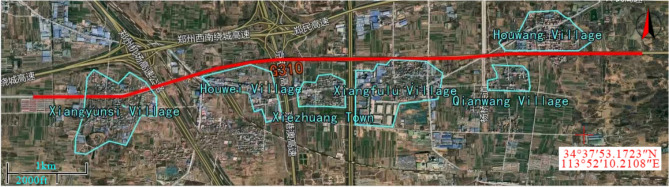
Satellite map of the survey area. Created by 91weitu, version number: v19.3.4, URL link: https://www.91weitu.com.

Based on the above sampling principles, the section of National Highway G310 from Xiangyunsi Village to Houwang Village in the suburbs of Zhengzhou City was selected as the survey area. Satellite map of the survey area was drawn using 91 weitu software, as shown in Fig. 1. To ensure the validity of the survey data, all surveys are conducted in good weather conditions and high visibility, the survey period selected for the morning peak of 7:00–9:00, the evening peak of 17:00–19:00. The survey considered the peak traffic periods and avoided Monday mornings as well as Friday evenings^12^.

### Data collection methods

**Figure 2:**
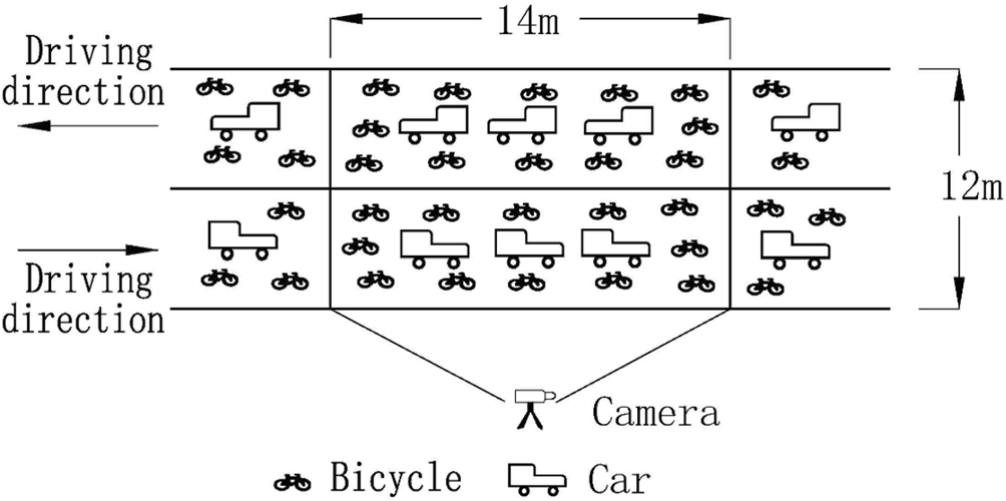
Virtual coil diagram.

The traffic survey data collection method uses "video filming + virtual coil method", and the main equipment includes a camera, infrared radar speed gun, tripod, stopwatch, range finder, etc. Its working principle is that, after selecting the survey position, two straight lines are delineated on the ground of the non-motorized vehicle driving area. The distance between the two straight lines is 14 m, and the width of the one-way lane is 6 m. Road cross-section of the same direction of travel between the motor vehicle lane and non-motorized lanes without isolation measures. The two straight lines are used as virtual detection coils, and the area is regarded as a video shooting detection area, as shown in Fig. 2. Each video is segmented according to a 30 s segment, and then Baidu AI's Flying Paddle EasyDL platform and Python are used to build a vehicle detection model and a vehicle speed detection model to extract the traffic volume and driving speed from the captured video, which is used as a basis to further calculate the flow rate and vehicle density of the detection area^12,15^.

#### Traffic volume

The built vehicle detection model is a deep learning-based target tracking model using Flying Paddle EasyDL^16^. The principle is to use mathematical methods such as the Kalman filtering algorithm and Hungarian algorithm to detect and identify specific motion objects in the video stream, to obtain the motion parameters and similarity of the target, to achieve motion prediction (trajectory, speed, etc.) and data matching for the object in each subsequent frame of the video.

#### Vehicle traveling speed

The speed detection model runs in the Python language environment. The principle is to track the rear of the vehicle in the video by target detection (assuming the camera is installed on a one-way street) and to calculate the pixel distance based on the detection frame spacing of two consecutive frames. Then the actual driving distance is obtained through pixel distance conversion. The travel time is calculated through the video frame rate, and then the driving speed is obtained and displayed in the video. The calculation formula is as in Eq. (1):

1 $$v=\frac{Vx}{ppm}\times fps\times 3.6$$

In Eq. (1), $v$–the speed of the vehicle when passing the virtual coil(m/s); $x$–vehicle displacement in two adjacent frames(m); ${t}_{1}$–the pixels contained in the video at a distance of 1 m, related to the frame width and height; $fps$–detecting the frame rate of the video(frame/sec).

#### Flow rate

Traffic volume extraction will be divided into 30 s segments for each video, and 30 consecutive 30 s video segments will be counted, which means the number of vehicles for 15 min will be counted. Different vehicle types are counted and converted into standard small car equivalent traffic volume, which is calculated as in Eq. (2):

2 $${q}_{x}={C}_{m}{q}_{m}+{C}_{n}{q}_{n}=\frac{{C}_{m}{n}_{m}}{t}+\frac{{C}_{n}{n}_{n}}{t}$$

In Eq. (2), ${q}_{x}$–total flow rate of mixed traffic flow(pcu/h); ${q}_{m}$, ${q}_{n}$–the respective flow rates of motor vehicles and non-motorized vehicles(pcu/h); ${n}_{m}$, ${n}_{n}$–the number of motorized and non-motorized vehicles passing the virtual coil during the observation time; ${C}_{m}$, ${C}_{n}$–the conversion factor for motorized and non-motorized vehicles is related to the proportion of the traffic flow in terms of size and type, with ${C}_{m}$ taking 1.25 and ${C}_{n}$ taking 0.815.

### Statistical sample size

The larger the capacity of the sample, the higher the accuracy will be. When the sample size obeys the normal distribution in the ideal state, the minimum sample size formula is as follows:

3 $$N\ge {\left(\frac{SK}{E}\right)}^{2}$$

In Eq. (3), $N$–minimum observation sample size; $S$–standard deviation of the sample; $T$–obeying the t-distribution statistic with degrees of freedom $n-1$, $K=1.96$ when the confidence level is 95% (90%); $E$–sample standard deviation size.

When the confidence level is 95%, $T$ is 1.96, the standard deviation of non-motorized vehicle speed is 4 km/h, the standard error is 1 km/h, and the minimum sample size is 64.

## Analysis of mixed traffic flow traffic characteristics

### Analysis of mixed traffic flow ratio

**Figure 3:**
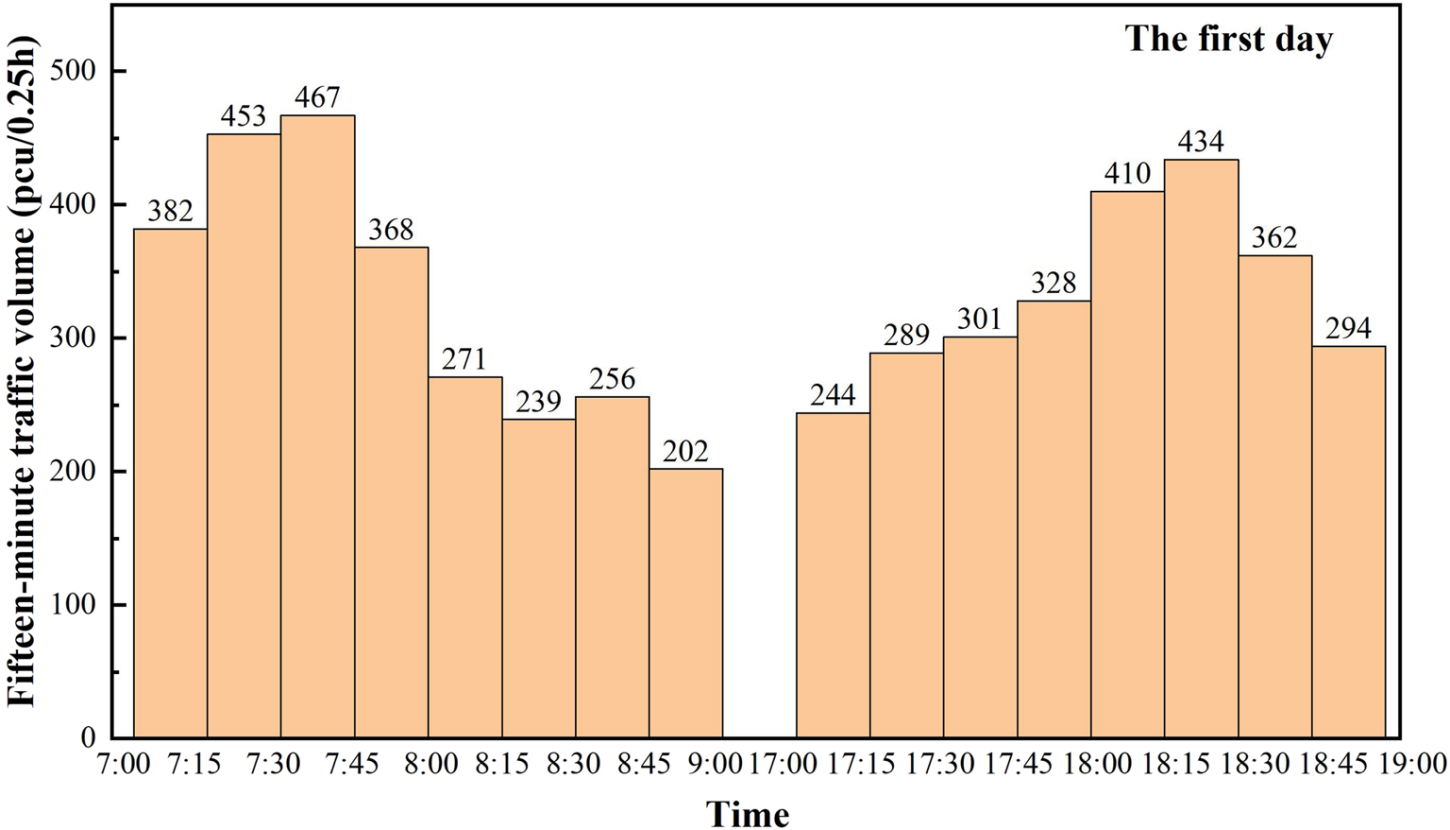
Mixed traffic counts for the first day peak traffic hours.

**Figure 4:**
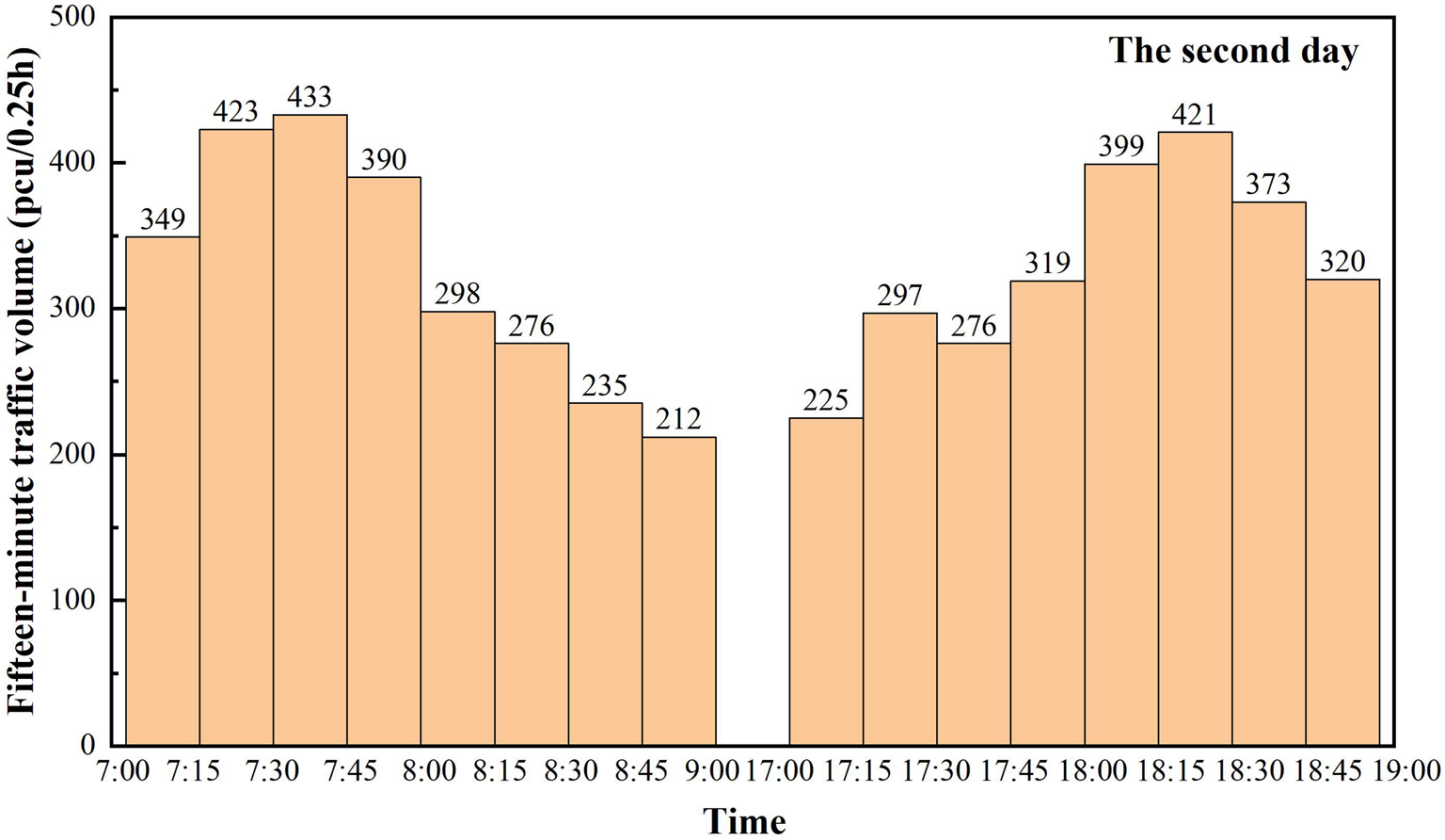
Mixed traffic counts for the second day peak traffic hours.

**Figure 5:**
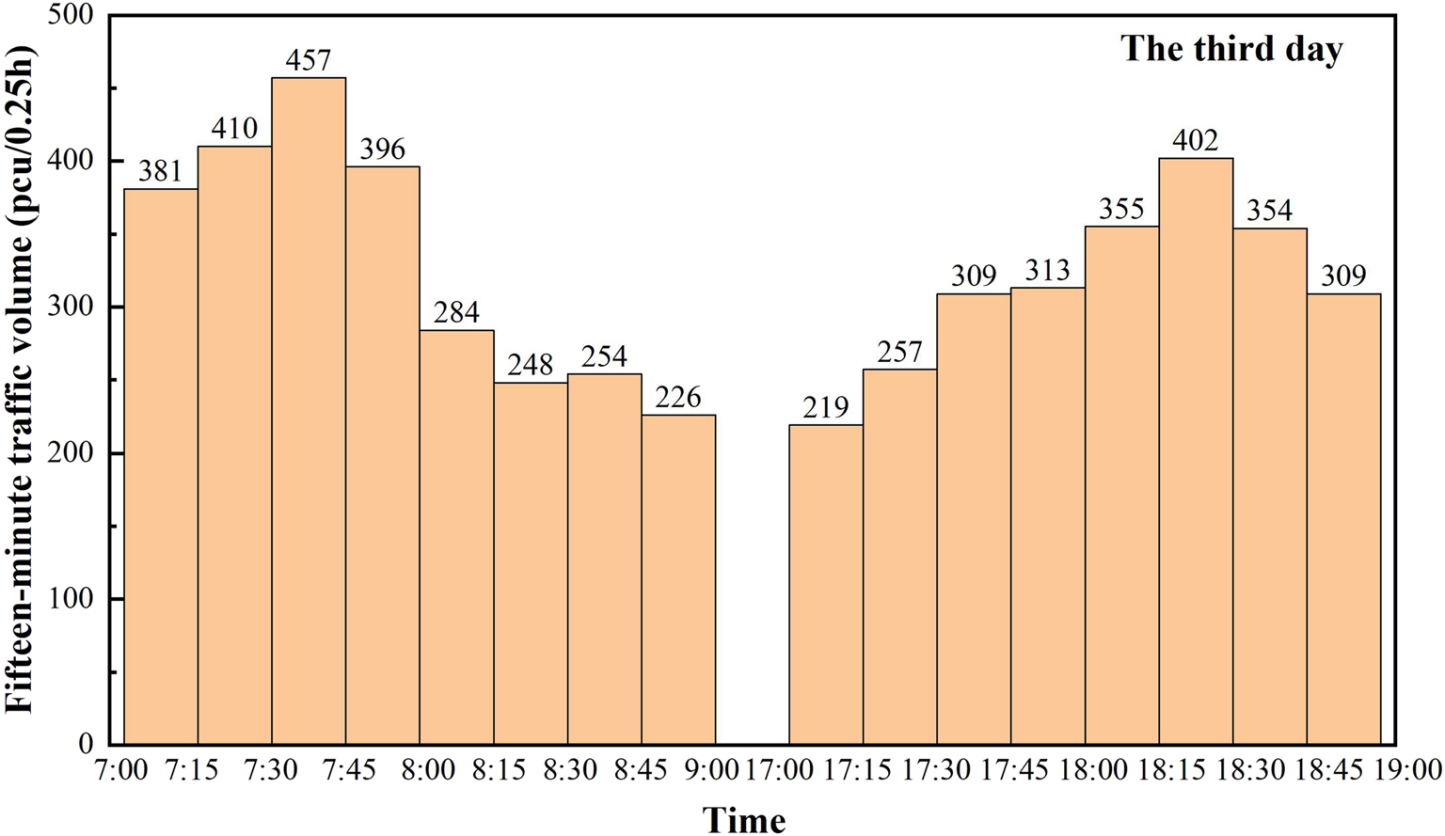
Mixed traffic counts for the third day peak traffic hours.

The section selected for investigation has the typical characteristics of a trunk highway through a small town section, which is a secondary road with a design speed of 60 km/h and a road width of 11.5 m, including a travel lane of 2 × 3.75 m and a shoulder of 2 × 2 m. The road crosses several small towns and villages, with a high number of non-motorized vehicles and a complex road traffic environment. The traffic survey lasted for three days, and according to reference^12^, it is known that due to the layout characteristics of small towns, a large number of non-motorized vehicles gather in some road sections during specific time periods. Therefore, the survey period is selected from 7:00–9:00 in the morning peak and 17:00–19:00 in the evening peak, and the statistical analysis can better reflect the characteristics of mixed traffic flow on this type of roadway. Traffic flow data were counted in 15-min increments, and Figs. 3, 4 and 5 reflect the traffic volumes at different times of the day over the three days, respectively.

**Figure 6:**
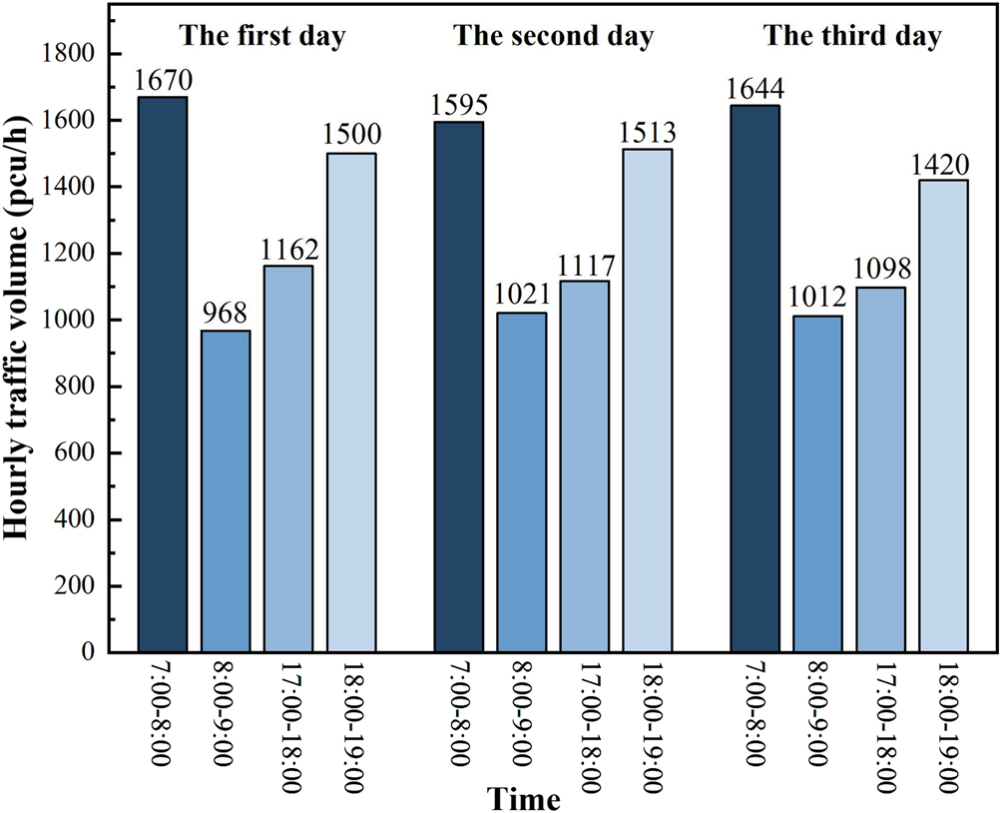
Statistical values of mixed traffic volumes during peak traffic hours.

Based on the statistical data of three days, a total of 15,720 vehicles are counted. Figure 6 is a combination of the statistical value of traffic volume per hour in the three days of peak time obtained from Figs. 3, 4 and 5, and there is a clear morning and evening traffic peak period. Due to the superposition of multiple travel demands, the traffic volume during the 7:00 am–8:00 am time period is the peak hourly traffic volume of the day, with hourly traffic volumes reaching 1670 pcu/h, 1595 pcu/h and 1644 pcu/h, respectively.

**Figure 7:**
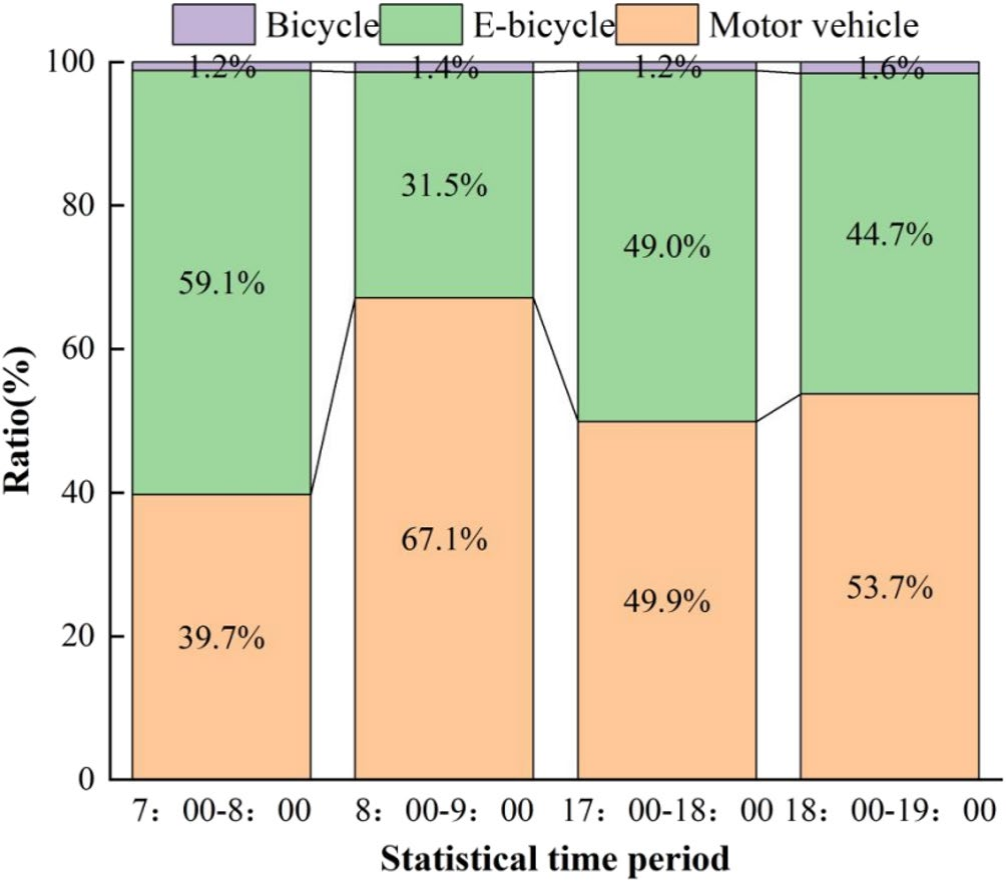
The ratio of the number of motorized vehicles to non-motorized vehicles in the traffic flow.

According to the actual traffic flow data of the four time periods, the average value of each time period in three days was taken to obtain the proportion of motor vehicle and non-motor vehicle traffic flow in the four time periods, as shown in Fig. 7. In terms of travel time, non-motorized vehicles accounted for the highest percentage in the morning from 7:00 to 8:00. In terms of the proportion of travel, the proportion of human-powered bicycles is very small and negligible, while the main traffic is motorized vehicles and electric bicycles, which account for more than 98% of the traffic. The maximum speed of electric bicycles is 25 km/h, which is very different from that of motor vehicles, and the mixture of motor vehicles and non-motorized vehicles can easily lead to traffic accidents.

### Analysis of mixed traffic flow speed

The average speed of a mixed traffic flow is the arithmetic average of the speeds of all vehicles passing through the detection area within a segment of the survey video. The data from the survey videos were collated and the average speed data for a total of 121 sets of mixed traffic flows were calculated. The overall speed of mixed traffic was statistically analyzed with a speed interval of 2 km/h, and the average speed distribution of mixed traffic flow was obtained, as shown in Table 1:

**Table 1:** Average speed distribution of mixed traffic flow on the surveyed section.

Speed interval/(km∙h^−1^)	Frequency/%	Cumulative frequency/%
(26, 28)	1.7	1.7
(28, 30)	5.7	7.4
(30, 32)	7.5	14.9
(32, 34)	10.7	25.6
(34, 36)	18.2	43.8
(36, 38)	21.5	65.3
(38, 40)	14.9	80.2
(40, 42)	8.2	88.4
(42, 44)	5.0	93.4
(44, 46)	3.3	96.7
(46, 48)	0.8	97.5
(48, 50)	2.5	100

**Figure 8:**
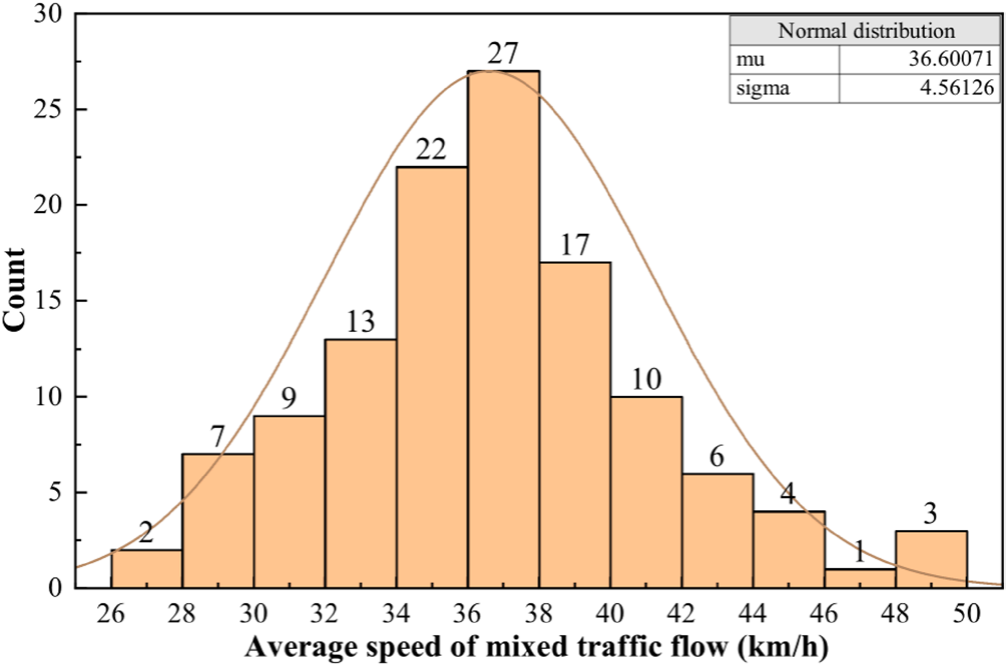
Average speed distribution of mixed traffic flow on the surveyed section.

According to Table 1, bar graphs can be drawn as shown below:

From Fig. 8, it can be seen that the average speed of mixed traffic flow of mainline highways through villages and towns is concentrated at 28 ~ 46 km/h. The distribution frequency is highest in two intervals of 34 ~ 36 km/h and 36 ~ 38 km/h, and gradually decreases along both sides of the x-axis. K-S tests were performed by SPSS and the results are shown in Table 2:

**Table 2:** Results of the one-sample K-S test for average vehicle speed.

Number of cases	Normal parameters		Test statistics	Asymptotic significance (two-tailed)
	μ	σ		
121	36.60071	4.561264	0.072	0.194

From the test results in Table 2, the average value of vehicle speed of mixed traffic flow is 36.6 km/h, and the most extreme difference with the standard normal curve is 0.072, which indicates that the change distribution curve overlaps well with the normal distribution curve. The asymptotic significance is greater than 0.05, indicating that the test result rejects the original hypothesis "the sample does not conform to normal distribution". This result shows that the average speed distribution pattern of mixed traffic flow of arterial highways through villages and towns conforms to normal distribution.

### Analysis of mixed traffic flow speed and flow rate

**Figure 9:**
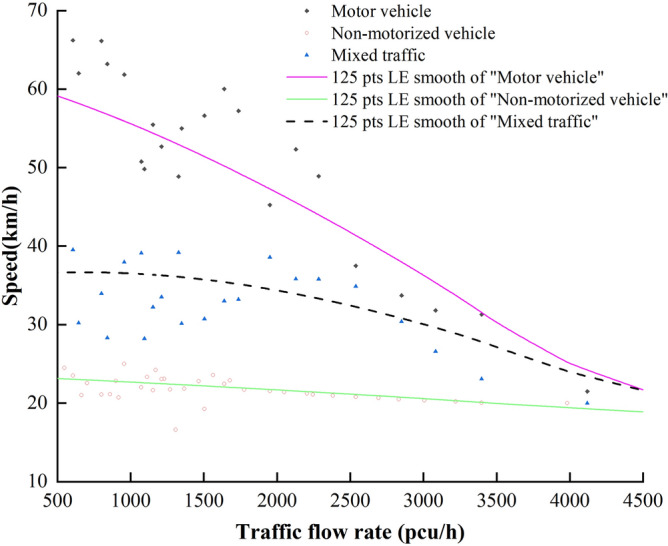
Average vehicle speed-flow rate correspondence.

In order to study the relationship between flow rate and speed of mixed traffic, the traffic flow data were counted in 15-min units to obtain the equivalent hourly flow rate (vehicles/0.25 h) of the surveyed road sections, and the survey data were arranged in descending order of flow rate by "video shooting + virtual coil method" to calculate the average speed of motor vehicles, non-motor vehicles and mixed traffic respectively. The average speed of motor vehicles, non-motorized vehicles and mixed vehicles was calculated. The data were smoothed and plotted on the dotted line graph, and the relationship between the average speed of traffic flow and the flow rate is shown in Fig. 9.

From Fig. 9, it can be seen that:

1. When the mixed traffic flow rate is low, the mutual interference between motor vehicles and non-motor vehicles running is small, the distribution of motor vehicle speed scatter is more dispersed, the vehicle speed dispersion is large, and the vehicle is in the free flow driving state. When the flow rate continues to increase, the interference between vehicles gradually strong, scattered distribution is more concentrated, and a downward trend, the average speed is reduced, the speed dispersion becomes smaller.
2. The average speed of motor vehicles decreases continuously with the increase of mixed traffic flow rate, and the average speed of non-motor vehicles fluctuates in a small range around 20 km/h. The average speed of mixed traffic flow decreases with the increase of traffic flow rate and finally tends to be stable.
3. When the mixed traffic flow rate is low, the speed of motor vehicles is much higher than that of non-motor vehicles, and the traffic flow speed is influenced by motor vehicles. When the mixed traffic flow rate increases, traffic congestion occurs, and motor vehicles can only follow non-motor vehicles to move, and the mixed traffic flow speed depends on the running speed of non-motor vehicles.

## Non-motorized crossing opening spacing

It can be concluded that the main highway through the small town section of motor vehicles and non-motorized vehicles mixed, easy to produce traffic congestion, traffic accidents, road capacity decline and other traffic problems, should be used to isolate the motor vehicle lane and non-motorized road space separation, and a reasonable determination of non-motorized road crossing opening spacing.

### Minimum crossing opening spacing based on main road traffic efficiency

Generally, vehicles will slow down to pass through the crossing openings and the passage time becomes longer. Therefore, when the length of the road section is certain, the more openings, the smaller the spacing between the crossing openings, the lower the road capacity. To better describe this relationship, the paper defines the capacity reduction rate $\tau$ of the road section to describe the impact of the crossing opening spacing on the main roadway capacity efficiency, which is calculated as shown in the following equation:

4 $$\tau =\frac{Travel \,time\, without\, crossing\, opening\, section(s)}{Travel\, time\, with\, crossing\, opening\, section(s)}$$

**Figure 10:**
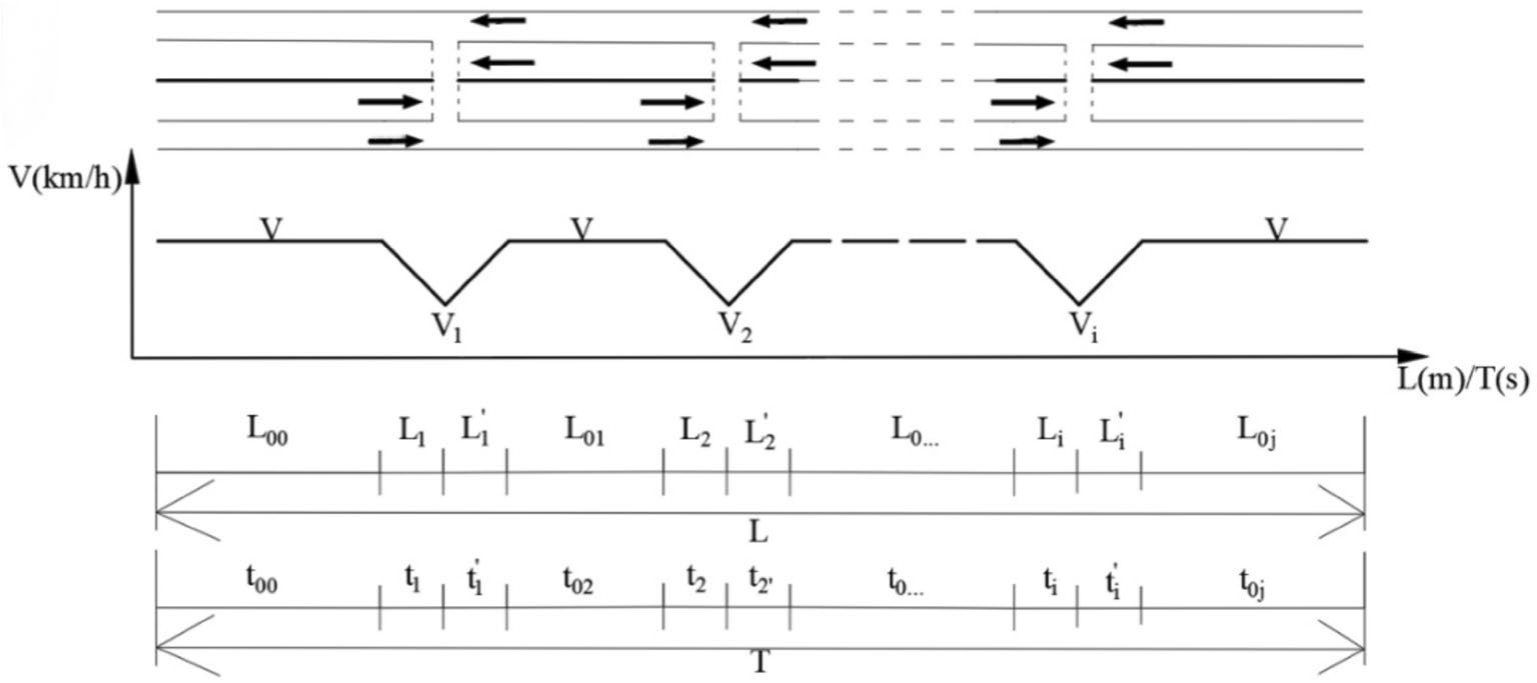
Road structure and speed change pattern diagram.

In the calculation process, the non-motorized crossing openings can be considered signal-free intersections where the main road priority principle is observed, and vehicles will slow down appropriately when passing through the non-motorized crossing openings. Assuming that the design speed of the vehicle is $V$, the vehicle travels at a uniform deceleration to ${V}_{i}$ when passing through the crossing opening $i$. When no non-motorized vehicles are present at the crossing opening, the vehicle only needs to slow down to ${V}_{i}$; when non-motorized vehicles are present, the vehicle needs to come to a complete stop. The sketch of the road structure and the law of speed change are shown in Fig. 10 below:

The capacity discount rate of a road section within a road section with n number of non-motorized crossing openings is calculated as follows:

5 $$ \tau  = \frac{{t_{{AB}} }}{{\sum\limits_{{j = 0}}^{n} {t_{{0j}} }  + \sum\limits_{{i = 1}}^{n} {\left( {t_{i}  + t_{i} ^{\prime }} \right)} }} = \frac{{\frac{L}{V}}}{{\sum\limits_{{j = 0}}^{n} {\frac{{L_{{0j}} }}{V}}  + \sum\limits_{{i = 1}}^{n} {\left( {\frac{{V - V_{i} }}{a} + \frac{{V - V_{i} }}{b}} \right)} }} = \frac{{\frac{L}{V}}}{{\frac{{L - \sum\limits_{{i = 1}}^{n} {\left(L_{i}  + L_{i} ^{\prime } \right)} }}{V} + \sum\limits_{{i = 1}}^{n} {\left( {\frac{{V - V_{i} }}{a} + \frac{{V - V_{i} }}{b}} \right)} }} $$

where, ${t}_{AB}$–the travel time when no crossing opening is set($s$); ${t}_{0j}$–the $j$ th section of uniform driving section of travel time($s$); ${t}_{i}$–the travel time of the upstream section of the $i$ th crossing opening($s$); ${t}_{i}{\prime}$–the travel time of the downstream section of the $i$ th crossing opening($s$); $L$–the length of the arterial highway through the village and town($m$); ${L}_{i}$–the length of the upstream section of the $i$ th crossing opening($m$); ${L}_{i}{\prime}$–the length of the downstream section of the $i$ th crossing opening($m$); $V$–the design speed of arterial highway($m/s$); ${V}_{i}$– minimum speed when passing the $i$ th crossing opening($m/s$); $n$–the number of non-motorized crossing openings in the road section; $a$–acceleration of the vehicle($m/{s}^{2}$); $b$–deceleration of the vehicle($m/{s}^{2}$).

Since the crossing opening specifications are the same, the vehicles in the ${L}_{1}$ and ${L}_{1}{\prime}$ sections are moving with uniform acceleration and uniform deceleration respectively. It is assumed here that ${V}_{1}={V}_{2}=\dots ={V}_{n}$, then Eq. (5) can be simplified as follows:

6 $$ \tau  = \frac{{\frac{L}{V}}}{{\frac{L}{V} - \frac{{n\left( {V^{2}  - V_{1}^{2} } \right)}}{{2aV}} - \frac{{n\left( {V^{2}  - V_{1}^{2} } \right)}}{{2bV}} + \frac{{n\left( {V - V_{1} } \right)}}{a} + \frac{{n\left( {V - V_{1} } \right)}}{b}}} = \frac{1}{{1 - n\left( {\frac{1}{{2aL}} + \frac{1}{{2bL}}} \right)\left( {V^{2}  - V_{1}^{2} } \right) + 2nV\left( {\frac{1}{{2aL}} + \frac{1}{{2bL}}} \right)\left( {V - V_{1} } \right)}} = \frac{1}{{1 + \frac{{nV^{2} }}{{2L}}\left( {\frac{1}{a} + \frac{1}{b}} \right)\left( {1 - \frac{{V_{1} }}{V}} \right)^{2} }} $$

Assuming that the spacing $d$ of adjacent non-motorized crossing openings within a fixed length $L$ section is equal, the relationship between the spacing $d$ and the number of openings $n$ can be obtained from Fig. 8 as follows:

7 $$d=\frac{L}{n+1}$$

Substituting Eq. (7) into (6), the relationship between the capacity reduction rate $\tau$ and the non-motorized crossing opening spacing $d$ can be obtained as:

8 $$\tau =\frac{1}{1+\frac{{V}^{2}}{2}\left(\frac{1}{d}-\frac{1}{L}\right)\left(\frac{1}{a}+\frac{1}{b}\right){\left(1-\frac{{V}_{1}}{V}\right)}^{2}}$$

where,$d$–spacing of adjacent non-motorized crossing openings($m$).

From Eq. (8), it can be seen that for a fixed-length section, the capacity reduction rate is proportional to the non-motorized crossing opening spacing $d$ and the speed reduction rate $\frac{{V}_{1}}{V}$ for motor vehicles. The smaller the opening spacing, the greater the number of crossing openings, so the number of vehicle decelerations increases, leading to an increase in travel time and a decrease in the reduction rate τ. The greater the speed reduction rate $\frac{{V}_{1}}{V}$, the smaller the vehicle deceleration through the intersection, the lower the section travel time, and the greater the reduction rate $\tau$.

According to the analysis of traffic survey data, the length of the road section is 1 km, different design speed and crossing opening spacing under the capacity reduction factor as shown in Table 3:

**Table 3:** Capacity discount factor for different design speeds and opening spacing.

Crossing opening spacing	80 (km/h)	60 (km/h)	40 (km/h)
50	0.1969	0.3180	0.6555
75	0.2742	0.4180	0.7456
100	0.3411	0.4960	0.8006
125	0.3996	0.5586	0.8378
150	0.4512	0.6098	0.8645
175	0.4971	0.6526	0.8846
200	0.5381	0.6989	0.9004
225	0.5749	0.7200	0.9130
250	0.6083	0.7470	0.9234
275	0.6386	0.7706	0.9320
300	0.6663	0.7915	0.9394
325	0.7007	0.8101	0.9457
350	0.715	0.8267	0.9511
375	0.7365	0.8416	0.9559
400	0.7565	0.8552	0.9602
450	0.7922	0.8787	0.9673
500	0.8233	0.8986	0.9731
600	0.8748	0.9300	0.9819
700	0.9158	0.9538	0.9883
800	0.9491	0.9726	0.9931
900	0.9767	0.9876	0.9969

**Figure 11:**
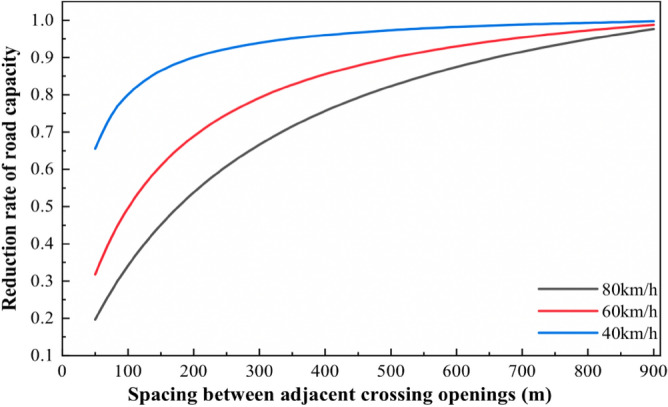
The relationship between crossing opening spacing and capacity reduction rate at different design speeds.

According to the data in Table 3, the relationship between the crossing opening spacing and the reduction rate of capacity at different design speeds is plotted as shown in Fig. 11:

Referring to the U.S. road level of service level index^17,18^, this paper selects the crossing opening spacing when the capacity discount rate is 0.7 as the minimum crossing opening spacing for non-motorized vehicles. At this time, the highway motor vehicle flow travels steadily with only slight delays, and the results are shown in Table 4 below:

**Table 4:** Minimum crossing opening spacing based on the efficiency of motor vehicle traffic.

Design speed (km/h)	80	60	40
Minimum crossing opening spacing (m)	325	200	100

### Maximum crossing opening spacing based on the psychological characteristics of cyclists

**Figure 12:**
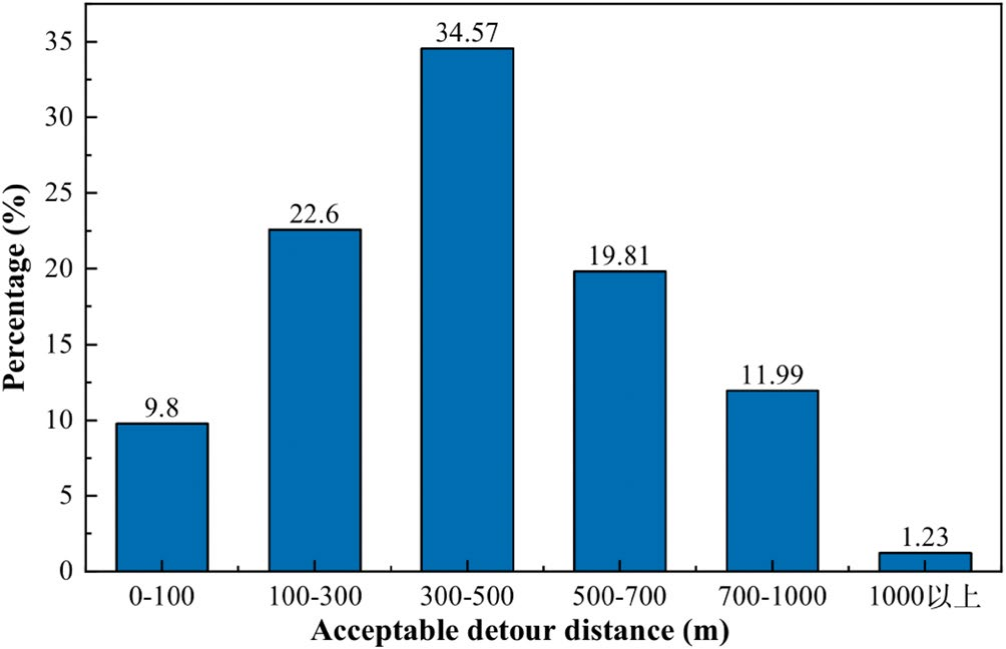
Distribution of acceptable detour distances for non-motorized street crossings.

The spacing of non-motorized crossing openings needs to be smaller than the acceptable detour distance for most cyclists to reduce the probability of non-motorized vehicles crossing the highway at random. The farthest acceptable crossing detour distance for non-motorized cyclists was counted by means of a questionnaire, as shown in Fig. 12:

From Fig. 12, it can be seen that more than 85% of the cyclists can accept a detour distance within 700 m. The highest percentage of cyclists, 34.57%, were able to accept a detour distance in the range of 300–500 m. Since the path of non-motorized vehicles in arterial highways has symmetrical characteristics when detouring from the crossing openings to their destinations, one-half of the acceptable detour distance is the maximum distance of crossing openings acceptable to cyclists. The questionnaire survey also found that the higher the design speed of the highway and the faster the motor vehicle travel speed, the less likely non-motorized cyclists will choose to cross the highway straight, and the greater the maximum acceptable crossing opening spacing for cyclists. Therefore, this paper combines the psychological characteristics of non-motorized cyclists to determine the maximum spacing of non-motorized openings under different highway classes, as shown in Table 5:

**Table 5:** Maximum crossing opening spacing based on the psychological characteristics of cyclists.

Design speed (km/h)	80	60	40
Maximum crossing opening spacing (m)	350	250	150

In summary, considering the efficiency of motor vehicle traffic and the psychological characteristics of cyclists, the range of values for the opening spacing of the side-split zone between motor vehicle lanes and non-motor vehicle lanes for arterial highway sections through small towns was obtained, as shown in Table 6:

**Table 6:** Appropriate interval for crossing opening spacing based on non-motorized demand.

Design speed (km/h)	80	60	40
Crossing opening spacing (m)	325 ~ 350	200 ~ 250	100 ~ 150

In the specific design, according to the design standard, the environment along the highway, in general, the higher the design standard of the highway, the larger the spacing between crossing openings; the denser the residents along the highway, the smaller the spacing between crossing openings.

## Simulation experiments and analysis

**Figure 13:**
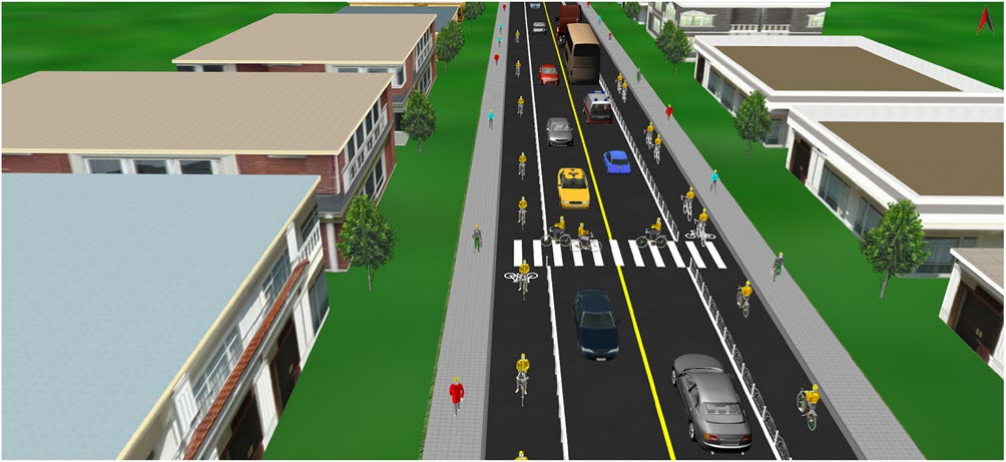
Schematic diagram of Vissim simulation model.

To verify the reliability of the calculation results, a simulation model was built using VISSIM software to simulate the traffic flow operation of the road under different crossing opening spacing, and the simulation results were verified with the above calculation results. Without considering the conditions of road elevation, slope, horizontal curvature, and other factors involving traffic control facilities such as signals, speed zones, etc., the modeling road is densely populated with residents on both sides and the traffic flow is high. The design speed is 60 km/h, the length is 1 km, the width of motorway is 3.5 m*2, and the width of non-motorway is 2.5 m. Since the non-motorized traffic flow is larger in the section of arterial highway through small towns, the non-motorized crossing opening width is taken as the larger value of 3 m. The non-motorized lane behavior mode is selected as free overtaking, and the default values are used for the rest of the road parameters. The established model is shown in Fig. 13:

### Simulation model parameter setting

According to the traffic survey results, 15,720 vehicles were counted for a total of 12 h in 3 days, and the average value of 1310 vehicles/h was taken as the hourly traffic volume of the setup. Meanwhile, the ratio of the number of motor vehicles and non-motor vehicles obtained from the survey for a total of 4 h a day respectively is taken as the average value as the ratio of the number of motor vehicles and non-motor vehicles in the simulation model according to Fig. 7. The speed distribution of the traffic flow and the proportion of vehicle types in the free-flow state are input into the model. The parameters of the simulation model are shown in Table 7:

**Table 7:** Simulation model parameters table.

Vehicle category	Motor vehicles	Non-motorized vehicles
Set traffic volume (pcu/h)	689	621
Proportion/%	52.6	47.4
Minimum expected speed (km/h)	36	10
Maximum expected speed (km/h)	63	28
85% bit expected speed (km/h)	49.6	20.3

### Simulation experimental design

The simulation experiment selects the number of crossings and spacing of openings as variables, and uses the control variable method to set up experimental groups for simulation. The number of openings and the spacing of openings are changed on a road of 1 km length and a crossover experimental group is set up. In addition, the number of openings is used as a univariate variable to set up the experimental group with uniform distribution of cross-street openings, and in this case, the opening spacing should be taken as the average of the length of the road section, as shown in Table 8. Since the simulation process has a certain randomness, 20 simulations were conducted for each experimental group changing the random seed and taking the average value as the final result.

**Table 8:** Mean distributed spacing experimental group.

Number of openings	19	16	13	11	10	9	8	7	6	5	4	3	2
Opening spacing (m)	50	60	75	90	100	112.5	128.5	150	180	225	300	450	900

### Analysis of simulation results

The simulation model built in Vissim was run to obtain the simulation results for the experimental group to obtain the travel time of a motor vehicle over a 1 km road for different opening spacing and the number of openings. Then analyze the change of the road capacity discount rate when the opening spacing and the number of openings are changed, and the simulation results are shown in Table 9:

**Table 9:** Road capacity reduction rate for different opening spacing and number of openings.

Number of openings	Opening spacing (m)							
	50	100	150	200	250	300	400	500
19	0.5206	–	–	–	–	–	–	–
16	0.5294	–	–	–	–	–	–	–
11	0.5545	0.5410	–	–	–	–	–	–
10	0.6266	0.5611	–	–	–	–	–	–
9	0.6414	0.5629	–	–	–	–	–	–
8	0.6620	0.5836	0.5729	–	–	–	–	–
7	0.6792	0.6094	0.5856	–	–	–	–	–
6	0.7012	0.6356	0.6126	0.5769	–	–	–	–
5	0.7203	0.6657	0.6450	0.6353	0.6458	–	–	–
4	0.7455	0.6978	0.6805	0.6730	0.6638	0.6802	–	–
3	0.7645	0.7334	0.7214	0.7144	0.7112	0.7115	0.7101	0.7247
2	0.7944	0.7713	0.7654	0.7621	0.7612	0.76	0.7608	0.7580

**Figure 14:**
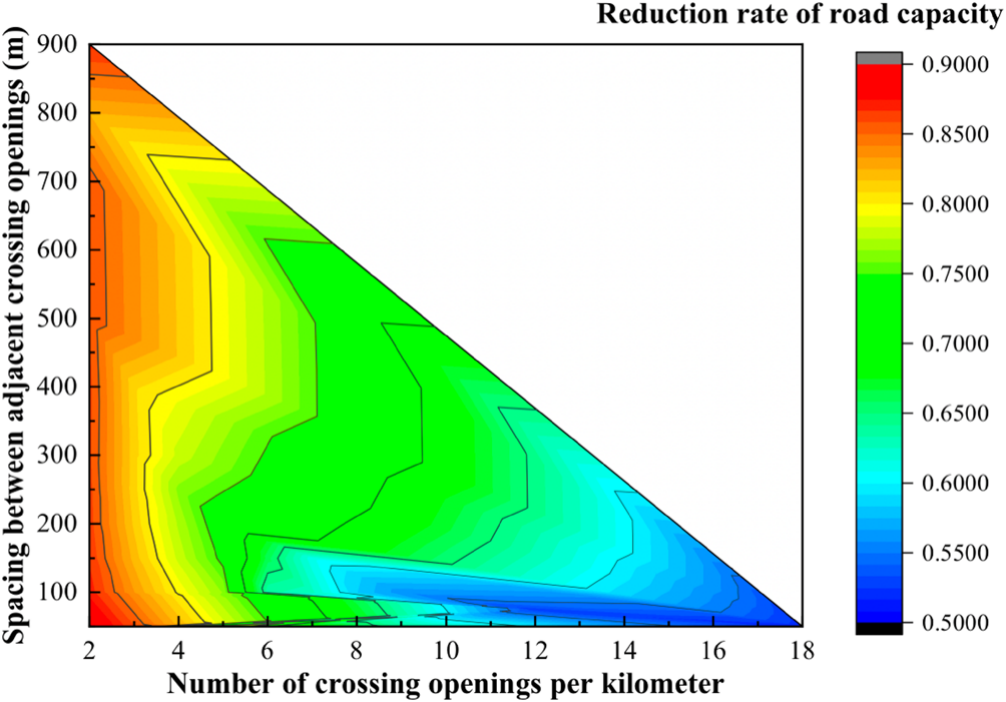
Contour map of road capacity reduction rate for different opening spacing and number.

**Figure 15:**
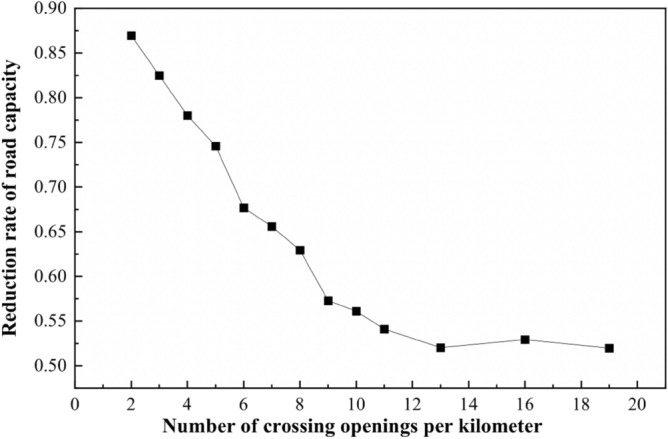
Number of crossing openings—capacity discount rate relationship line graph.

**Figure 16:**
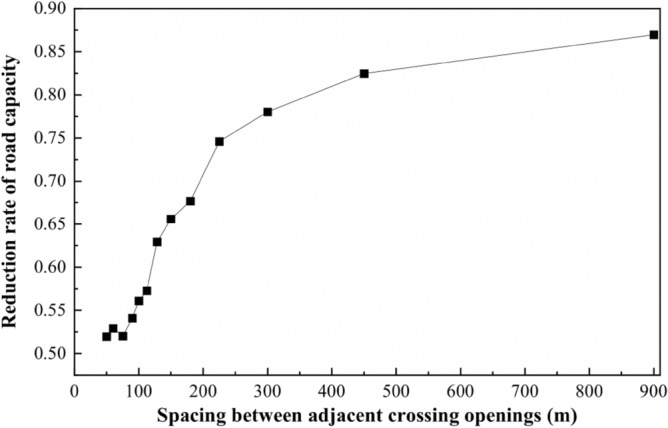
Opening spacing—capacity discount rate relationship line graph.

The simulation results were converted to visual graphics and the results are shown in Fig. 14:

In Fig. 14, when the spacing of the crossings is constant, the road capacity discount rate decreases with the increase of the number of openings; when the number of crossings is constant, the road capacity discount rate does not change significantly. It can be seen that the road capacity discount rate is mainly affected by the number of crossing openings. According to Table 9, the relationship between the road capacity discount rate and the number of crossing openings when the road is divided completely equidistant, is shown in Fig. 15:

According to the relationship between the spacing of openings and the number of crossing openings, the relationship between the spacing of openings and the discount rate of capacity can be obtained, as shown in Fig. 16:

In Fig. 16, when the spacing between openings is about 200 m, the capacity reduction rate is about 0.7. When the spacing is less than 200 m, the capacity discount rate decreases sharply, and the traffic operation condition is poor. Therefore, when the road design speed of 60 km/h, the non-motorized crossing opening spacing is set in the range of 200 ~ 250 m or so best, consistent with the results of the analysis above.

## Discussion

The setting of side dividers between motorized and non-motorized lanes on arterial highway sections through small towns is an effective way to solve many traffic problems. However, how to set up reasonably spaced non-motorized crossing openings while ensuring the efficiency and safety of highway traffic is the key issue that needs to be solved nowadays. Based on the mixed traffic characteristics of China's arterial highways passing through small towns, this paper studies the spacing of non-motorized vehicles crossing the street openings in combination with the entrance and exit management technology. To a certain extent, it fills the research gap, which not only helps to improve the traffic efficiency and safety level of the trunk highway, but also can be used as the theoretical support for the current non-motorized vehicle traffic management and cycling space–time planning.

Compared with the existing research results^19–21^, many experts and scholars focus on the management of driving speed, which is used to improve road traffic safety and reduce the occurrence of accidents. The research conclusions are not fully applicable to the arterial highway crossing the urban section, and have great limitations. Taking Sweden as an example, the speed limit for secondary roads crossing villages and towns is 30 km/h. European traffic accident report shows that 25 people died in traffic accidents in an average of 1 million residents in Sweden. Combined with the research results of this paper, if the spacing of crossing openings is too small, it will lead to frequent deceleration of motor vehicles on the main road and increase delay. If the spacing is too large, it will increase the cyclist's detour distance, resulting in some non-motor vehicles crossing the highway at will, interfering with the normal driving of motor vehicles, and even causing traffic accidents. Therefore, it is necessary to comprehensively consider the traffic efficiency of motor vehicles and the psychological characteristics of cyclists, and set an appropriate range for the spacing of non-motor vehicles crossing the street. Due to the limitation of conditions, the survey data in this paper are only from the plain area of China. For other countries, due to the differences in traffic laws and regulations, traffic flow characteristics and actual terrain, the recommended value of cross-street opening spacing given in this paper may not be applicable to all countries, but for other countries with both secondary roads and major roads crossing small towns. It provides a reference for the research ideas that can be used for reference, and provides a reference for the trade-off between improving road safety and ensuring traffic efficiency.

## Conclusions

The main body of traffic on the arterial highway through the small town section is motor vehicles and electric bicycles. In terms of travel time periods, non-motorized vehicles have the highest share during the 7:00–8:00 a.m. time period, which generates the daily peak hour traffic volume. The speed of mixed traffic flow is normally distributed, and the driving speed is concentrated within 28 ~ 46 km/h. The percentage of non-motorized vehicles has an important influence on the average speed of mixed traffic flow, and the driving speed of motorized vehicles decreases continuously with the increase of mixed traffic flow rate.

From the two dimensions of road traffic efficiency and the psychological characteristics of cyclists, the setting range of non-motorized lateral crossing opening spacing was calibrated under different design standards: when the road design speed is 40, 60 and 80 km/h, the recommended range of opening spacing is 100 ~ 150 m, 200 ~ 250 m and 325 ~ 350 m respectively. Through simulation experiments, the opening spacing range calibrated in this paper is verified, and it is proved that it can achieve good expectations in actual engineering, effectively improve road capacity and reduce the risk of road accidents.

In practical engineering, it is still necessary to consider the development intensity of land along the highway. In the future, the scope of investigation and research can be expanded, and suggestions can be given for the setting of the horizontal crossing opening spacing value of the trunk highway crossing the small town section in different terrain environments.

## Data Availability

The authors confirm that the data supporting the findings of this study are available within the article.
